# Organoids and spheroids: advanced *in vitro* models for liver cancer research

**DOI:** 10.3389/fcell.2024.1536854

**Published:** 2025-01-09

**Authors:** Mirella Pastore, Alessia Giachi, Elena Spínola-Lasso, Fabio Marra, Chiara Raggi

**Affiliations:** Department of Experimental and Clinical Medicine, University of Florence, Florence, Italy

**Keywords:** 3D culture, organoids, spheroids, liver cancer, drug-screening

## Abstract

Liver cancer is a leading cause of cancer-related deaths worldwide, highlighting the need for innovative approaches to understand its complex biology and develop effective treatments. While traditional *in vivo* animal models have played a vital role in liver cancer research, ethical concerns and the demand for more human-relevant systems have driven the development of advanced *in vitro* models. Spheroids and organoids have emerged as powerful tools due to their ability to replicate tumor microenvironment and facilitate preclinical drug development. Spheroids are simpler 3D culture models that partially recreate tumor structure and cell interactions. They can be used for drug penetration studies and high-throughput screening. Organoids derived from stem cells or patient tissues that accurately emulate the complexity and functionality of liver tissue. They can be generated from pluripotent and adult stem cells, as well as from liver tumor specimens, providing personalized models for studying tumor behavior and drug responses. Liver organoids retain the genetic variability of the original tumor and offer a robust platform for high-throughput drug screening and personalized treatment strategies. However, both organoids and spheroids have limitations, such as the absence of functional vasculature and immune components, which are essential for tumor growth and therapeutic responses. The field of preclinical modeling is evolving, with ongoing efforts to develop more predictive and personalized models that reflect the complexities of human liver cancer. By integrating these advanced *in vitro* tools, researchers can gain deeper insights into liver cancer biology and accelerate the development of novel treatments.

## Introduction

Primary liver cancers, notably cholangiocarcinoma (CCA) and hepatocellular carcinoma (HCC) encompass a heterogeneous group of malignancies that present significant clinical challenges. These cancers are often characterized by a lack of specific biomarkers and frequently manifest as asymptomatic in their early stages, resulting in delays in diagnosis and adverse prognoses (Valle et al., 2021; Tsung et al., 2024; Banales et al., 2020). CCA is further categorized based on its anatomical location within the biliary tree, which includes intrahepatic (iCCA), perihilar (pCCA), and distal (dCCA) forms (Ilyas et al., 2018). Currently, surgical resection is the only curative treatment available for these primary liver cancers, underscoring the urgent need for effective pharmacological interventions (Orcutt and Anaya, 2018).

In this landscape, advanced *in vitro* three-dimensional (3D) culture models have emerged as crucial tools for enhancing our understanding of liver cancer biology. These sophisticated models allow for a more accurate representation of the tumor microenvironment, facilitating the exploration of molecular mechanisms, identification of new therapeutic targets, and expedited yet reliable screening of potential novel drugs (Nuciforo and Heim, 2021; Blidisel et al., 2021). Additionally, patient-derived organoids (PDOs) are gaining prominence as innovative platforms for developing personalized treatment strategies.

This review highlights recent studies using 3D models to assess drug responses and advance treatment options for liver cancer. By focusing on their integration into preclinical research, it underscores their significance in discovering and developing effective therapies.

## 3D *in vitro* models in primary liver cancer

### Spheroids in hepatocellular carcinoma: insights into cancer biology and therapeutic strategies

Since the 1970s, tumor spheroids have been employed to simulate tumor biology, forming 3D multicellular aggregates primarily derived from two-dimensional (2D) cancer cell cultures and occasionally including stromal components such as endothelial cells and fibroblasts (Inch et al., 1970; Shoval et al., 2017; Österholm et al., 2012). These spheroids self-assemble using anchoring-independent culture methods or scaffold systems like Matrigel droplets (Calvisi et al., 2023; Jubelin et al., 2022). A significant advantage of spheroids over 2D cultures is their ability to maximize cell-to-cell interactions and replicate the gradients of oxygen and drug transport found within tumors (Habanjar et al., 2021).

In HCC, the existence of a subpopulation of cancer cells with stem-like characteristics is well documented (Yamashita et al., 2009). Tumor spheroids are particularly useful for investigating potential stemness markers in HCC that may serve as targets for anti-cancer stem cell therapies (Wang YY. et al., 2024; Roy et al., 2024).

Sorafenib, a multi-tyrosine kinase inhibitor, is the first targeted therapy approved for HCC. Although it exhibits significant anticancer and anti-angiogenic effects, some patients develop resistance (Kong et al., 2021). Recent studies have created spheroids from sorafenib-resistant HuH7 cell lines to evaluate alternative treatments in a fibrotic microenvironment (Sariyar and Karagonlar, 2023; Sariyar et al., 2023). In their work, Sarıyar et al. noted a significant reduction in the CD133-positive stem cell population and an increase in CD24 and EpCAM-positive cells in sorafenib-resistant spheroids, suggesting that these markers may contribute to drug resistance. They tested new drugs, Gefitinib (an EGFR inhibitor) and PP2 (a Src-family kinase inhibitor), finding that their combination was more effective in inducing cell death in resistant spheroids compared to single treatments (Sariyar et al., 2023).

To assess the toxicity of anti-HCC therapies while preserving healthy liver tissue, Royo et al. developed 3D spheroids from both HCC cells (HEPG2 and HuH7) and healthy liver cells. Treatments with standard anti-HCC drugs (Dacarbazine, Methotrexate, Sorafenib) revealed a marked decrease in tumor cells, with Sorafenib showing the strongest impact. The study also tracked liver-derived extracellular vesicles as indicators of hepatocyte damage, revealing that spheroid treatments increased vesicle release, thereby providing a dual approach to evaluating drug efficacy and toxicity (Royo et al., 2024).

Anti-PD-1 immune checkpoint inhibitors (ICIs) are approved systemic therapies for HCC (Sankar et al., 2024). However, patients with mutated β-catenin often have poor outcomes (Akasu et al., 2021). A recent study utilizing HCC-derived spheroids explored the role of β-catenin in immune evasion, showing that silencing β-catenin enhanced the infiltration of peripheral blood mononuclear cells (PBMCs) into spheroids (Dantzer et al., 2024). Conversely, treatment with CHIR-99021, a GSK3β inhibitor, reduced immune infiltration, indicating a possible mechanism by which β-catenin aids tumor escape from immune surveillance.

In efforts to enhance NK cell-mediated tumor responses, a recent study introduced antibody-based therapies known as NK cell engagers (NKCEs), specifically targeting Glypican-3 (GPC3) in HCC (Arulanandam et al., 2023). The addition of CYT-303, an NKCE that binds both NK cells and GPC3, significantly augmented the cytotoxic effects of peripheral blood-derived NK cells on Hep3B spheroids in a dose-dependent manner, offering a promising avenue for immunotherapy in HCC.

Investigating the effects of CHIR-99021 on stromal cells, one study developed spheroids composed of HCC cells and hepatic stellate cells (HSCs) (Song et al., 2024). Given the connection between liver fibrosis and HCC, these mixed spheroids demonstrated increased expression of epithelial-mesenchymal transition (EMT) markers. Treatment with CHIR-99021 reduced these markers and highlighted the potential for antifibrotic strategies in HCC therapy. Table 1 summarizes the drugs tested and their targets identified through HCC spheroid research.

**TABLE 1:** Overview of disease types, experimental models, target biomarkers and therapeutic agents.

Disease	Model	Drug	Target/Biomarker	Ref.
HCC	HepG2 spheroids	4µ8C plus Doxorubicin	ER-stress	Kopsida et al. (2024)
		Talazoparib	—	Zhang et al. (2024a)
	Hep3B spheroids	NK cell engager CYT-303	GPC3	Arulanandam et al. (2023)
	HuH7 spheroids	—	SERPINE2	Zhang et al. (2024b)
	HepG2 and HuH7 spheroids	—	TESC	Ye et al. (2024)
	Hep3B, HuH7 and SK-Hep1 spheroids	Prasugrel	USP1	Bian et al. (2024)
	HuH7 and Mahlavu spheroids	G4 stabilizer RHPS4 plus Sorafenib	CTC1	Kipcak et al. (2024)
	HuH7 and SNU449 spheroids	—	lncRNA-KCNQ1OT1	Majumdar et al. (2024)
	HepG2 or HuH7 and HSCs heterospheroids	Benja-ummarit	Ferroptosis	Sandech et al. (2024)
	HuH7 and HSCs heterospheroids	Gefitinib plus PP2	EGFR and Lyn	Sariyar et al. (2023)
		CHIR-9901	DNMT3B	Song et al. (2024)
	Murine PDOs	—	HKDC1	Fan et al. (2024)
	Murine NAFLD-associated PDOs	Supplementation of *Lactobacillus acidophilus*	Prophylaxis	Lau et al. (2024)
	Murine and human PDOs	—	YTHDF1	Zhang et al. (2024c)
	Human PDOs	Proteasome inhibitors plus Dinaciclib	Proteasome and CDK	Lim et al. (2022a)
		Atezolizumab	PD-L1	Zou et al. (2023)
		4µ8C plus Doxorubicin	ER-stress	Kopsida et al. (2024)
		Talazoparib	—	Zhang et al. (2024a)
		—	SERPINE2	Zhang et al. (2024b)
		Kpt185	XPO1	Yang et al. (2024b)
		Cpd-63	PTPRE	Dong et al. (2024)
		—	FDX1	Sun et al. (2024b)
		Erastin	Ferroptosis	Li et al. (2024b)
		—	WDR20	Jiang et al. (2024)
		—	SLC25A15	Zhang et al. (2024d)
		—	METTL16	Wang et al. (2024b)
		SAHA or AZD5363 plus Lenvatinib	HDAC or AKT	Yan et al. (2024)
		—	MRPL12	Ji et al. (2024)
		—	MCB1	Xiang et al. (2024)
		ABCB1 inhibitors plus Doxorubicin	ABCB1	Blukacz et al. (2024)
HB	Human PDOs	—	EGR1	Pan et al. (2024a)
CCA	KKU-M213 spheroids	Ceritinib	ALK	Myint et al. (2024)
	KKU-M213 or KKU-M156 and hCAFs heterospheroids	Crenigacestat	γ-secretase	Mancarella et al. (2024)
	HUCCT1 or SNU1079 and HSCs, fibroblasts, and endothelial cells heterospheroids	siRNA-tMNVs and RNP-tMNVs	PD-L1	Gondaliya et al. (2024)
	Human PDOs	NTRC 0652-0	Lck	Conboy et al. (2023)
		RPS6-V-PMO	RPS6	Fu et al. (2024)
		GSK3326595	PRMT5	Elurbide et al. (2024)
		—	PYGB	Pan et al. (2024b)
		Surufatinib plus photodynamic therapy	GPX4 and ACSL4	Huang et al. (2024a)
		Irinotecan plus Cisplatin	—	Rao et al. (2024)
		KRIBB-11	HSF1	Cigliano et al. (2024)
		Sarizotan or Sarizotan plus Cisplatin	MAL2	Huang et al. (2024b)
		VDAC1 antagonist	VDAC1	Conti et al. (2024)
		M2698 plus Dasatinib	S6K/AKT	Luk et al. (2024)
		Icaritin plus Gemcitabine plus Cisplatin	-	Kang et al. (2024)

ACSL4, Acyl-CoA synthetase long-chain family member 4; ALK, Anaplastic lymphoma kinase; CDK, Cyclin-dependent kinase; CTC1, Conserved Telomere Maintenance Component 1; DNMT3B, DNA methyltransferase 3B; EGFR, Epidermal growth factor receptor; EGR1, early growth response 1; FDX1, Ferredoxin 1; GPC3: Glypican-3; GPX4: Glutathione peroxidase 4; HDAC, Histone deacetylase; HKDC1, Hexokinase domain containing 1; HSF1, Heat Shock Factor 1; Lck, Tyrosine-protein kinase Lck; Lyn, Tyrosine-protein kinase Lyn; MAL2, T cell differentiation protein 2; MCB1, Multiubiquitin chain-binding protein 1; MNVs, Milk-derived nanovesicles; MRPL12, Mitochondrial ribosomal protein L12; PD-L1, Programmed death ligand 1; PRMT5, Protein arginine-methyltransferase 5; PTPRE, Protein tyrosine phosphatase receptor epsilon; PYGB, Glycogen phosphorylase brain form; RNP, ribonucleoprotein; RPS6, Ribosomal protein S6; RPS6-V-PMO, Phosphorodiamidate morpholino oligomer; SERPINE2, Serpin family E member 2; siRNA, small interfering RNA; SLC25A15, Solute carrier family 25 member 15; TESC, Tescalcin; USP1, Ubiquitin-specific protease 1; VDAC1, Voltage-dependent anion-selective channels 1; WDR20, WD repeat-containing protein 20; YTHDF1, YTH domain family 1.

Spheroids derived from HCC cells exhibit complex architectures that enhance the reliability of 3D models, especially when incorporating stromal components. Co-culturing with immune cells like T and NK lymphocytes offers efficient platforms for evaluating novel therapies aimed at boosting anti-tumor immunity. These developments underscore the importance of 3D models in advancing our understanding and treatment of liver cancers.

### 3D spheroid models for cholangiocarcinoma research

Similar to HCC, spheroids have gained prominence in CCA, where they effectively mimic the low oxygen levels present in tumor environments (Vanichapol et al., 2015). Metabolomic studies have demonstrated that iCCA spheroids exhibit altered metabolic profiles, including heightened glucose consumption and lactate excretion, indicative of a glycolytic shift (Ciufolini et al., 2024). Furthermore, analyses of various iCCA cell lines confirmed that spheroids display diminished antioxidant capacity and increased oxidative stress (Phukhum et al., 2023). These metabolic alterations enhance the relevance of iCCA spheroid cultures as models for studying anaerobic metabolism and tumor stress.

The 3D structure and metabolic changes observed in iCCA spheroids contribute to an enriched stem gene expression profile, significantly enhancing their tumorigenic properties compared to 2D cultures (Raggi et al., 2017). Consequently, spheroids are frequently used to evaluate novel treatments for CCA (Marin et al., 2019).

Recent advancements include the development of novel 3D heterospheroids composed of human cancer-associated fibroblasts (CAFs) and iCCA cells (Mancarella et al., 2024). CAFs are known to facilitate CCA progression through extracellular matrix deposition and interaction with malignant cells (Carloni et al., 2022). Studies have shown that these heterospheroids enhance iCCA cell proliferation and invasion. Notably, treatment with Crenigacestat, a γ-secretase inhibitor, reduced the viability and invasion of hCAF-iCCA heterospheroids (Mancarella et al., 2024), highlighting the potential for targeting stromal interactions in therapeutic strategies.

Mesenchymal stromal cells (MSCs) are emerging as potential components in the tumor microenvironment of iCCA, as they can contribute to liver fibrosis and differentiate into CAFs (Haga et al., 2015; Gan et al., 2021; Russo et al., 2006). The concept of MSCs pertains to a subset of non-hematopoietic cells found in the stromal bone marrow that are multipotent and possess the ability to self-renew. Recently, this definition has broadened to include cells originating from any connective tissue that can produce various types of stromal cells. MSCs also circulate in the bloodstream and can migrate to sites of inflammation (Dominici et al., 2006; Bianco et al., 2013; Ridge et al., 2017). Recent research demonstrated that adding MSCs to spheroids derived from patient-derived xenograft (PDX) models could restore lost signaling pathways, indicating their dual role in either promoting or inhibiting tumor growth. This emphasizes the importance of stromal elements in CCA modeling (Sueca-Comes et al., 2024).

The past decade has seen an increased exploration of ICIs in cancer, including anti-PD-L1 therapies approved for various cancers and CCA (Fiste et al., 2021). Recent studies utilized iCCA spheroids to test RNA-based anti-PD-L1 therapies, revealing that multicellular spheroids better mimic the tumor microenvironment and can effectively assess immunomodulatory responses (Gondaliya et al., 2024). Table 1 summarizes the drugs tested and their targets identified through CCA spheroid research.

3D *in vitro* models, particularly tumor spheroids, offer enhanced insights into CCA biology and the tumor microenvironment, proving essential for developing new therapeutic strategies. Their ability to incorporate stromal components and accurately reflect metabolic and immune interactions makes them invaluable for preclinical cancer research.

### Liver patient-derived organoids

The concept of organoids emerged in 2009, originating with the development of 3D cultures that mimic the structure and function of human organs, initially focusing on intestinal organoids. This foundational research paved the way for liver organoids, providing insights into liver tissue regeneration and early-stage diseases, and eventually extending to liver cancer models (Sato et al., 2009; Huch et al., 2013; Takebe et al., 2013). PDOs are 3D cultures derived from tumor tissues that maintain the architecture and heterogeneity of the original tumors. They are typically sourced from surgically resected tissues or needle biopsies, allowing for minimal tissue use and timely sample collection (Nuciforo and Heim, 2021; Nuciforo et al., 2018; Thorel et al., 2024).

PDOs are cultured in specialized matrices, such as Matrigel, with nutrient-rich media, preserving the histological and genetic characteristics of the parent tumor (Broutier et al., 2017). A recent biobank of liver cancer PDOs includes 44 HCC, 5 hepatoblastoma (HB), 12 iCCA, and 4 mixed HCC-CCA PDOs (Ji et al., 2023). Comprehensive genomic, epigenomic, transcriptomic, and proteomic analyses identified four molecular subtypes of liver cancer PDOs: L-LM (best prognosis), L-PL (poor prognosis, high proliferative signals), L-ICC (RAS signaling), and L-DM (altered drug metabolism) with distinct drug responses.

High-throughput screening revealed general sensitivity to TOP2 inhibitors, HDAC inhibitors, and BET PROTAC inhibitors while uncovering subtype-specific responses, L-PL showed high sensitivity to PI3K pathway inhibitors, while L-DM exhibited sensitivity to FGFR inhibitors. Studies indicated a relationship between Lenvatinib resistance and EGFR expression, and predictive models based on PDO proteogenomic data identified potentially effective drug combinations, such as Lenvatinib plus Temsirolimus (a mTOR inhibitor).

A recent report established long-term PDO cultures from 66 liver cancer patients, achieving a 40.9% success rate. This involved a two-step digestion method to minimize fibrotic tissue and utilized different media conditions for initiation and passaging. Drug screening from these PDOs yielded a successful treatment regimen for a diagnosed iCCA patient, highlighting the predictive potential of PDOs (Rao et al., 2024).

Studies employing pharmacogenomic profiling of liver cancer PDOs revealed significant intra-tumor heterogeneity, complicating treatment responses. Screening over 100 patients provided insights into drug sensitivities, revealing a cumulative sensitivity of 73% to seven targeted therapies, yet only 37.1% of patients benefited from monotherapy. Transcriptomic analysis identified 254 genes associated with Lenvatinib sensitivity, and a machine-learning approach yielded a panel of predictive biomarkers (Yang H. et al., 2024).

Additional research using PDOs and xenografts assessed a panel of 80 drugs to identify alternatives for Lenvatinib resistance. Key candidates included Romidepsin (an HDAC inhibitor), which displayed consistent effectiveness and enhanced immune responses when combined with anti-PD1 therapy (Sun L. et al., 2024). In a study focusing on the Chinese population, 64 organoid lines were evaluated for genomic and transcriptomic profiles, identifying variable genes and enrichment in pathways related to proliferation, resistance mechanisms, and immune evasion; this research emphasized the role of PDOs in predicting drug efficacy (Zhu et al., 2024).

As interest in PDOs for drug testing grows, numerous recent studies have aimed to evaluate the effectiveness of new therapeutic agents using these models. Table 1 summarizes the drugs tested and their targets identified through PDO research.

### Hepatocellular carcinoma patient-derived organoids (HCC-PDOs): challenges and advances

The establishment of HCC-PDOs has been particularly challenging due to factors such as low success rates, difficulties in developing them from well-differentiated specimens, larger necrotic areas, the predominance of healthy cells over malignant ones, and the heterogeneous nature of HCC tumors (Broutier et al., 2017; Sun L. et al., 2024; Li K. et al., 2024; Airola et al., 2024; Zhang et al., 2023). Despite these challenges, successful cultivation of HCC-PDOs has demonstrated their ability to accurately recapitulate tumor biology, thus representing a substantial advancement in disease modeling and providing valuable tools for identifying therapeutic targets and biomarkers.

PDOs may help maximize the application of drugs that have shown promise in preclinical studies but failed in clinical settings. For instance, a study by Lim et al. screened 268 drugs in PDOs derived from HCC-PDX and identified three proteasome inhibitors (Bortezomib, Carfilzomib, Ixazomib) and one CDK inhibitor (Dinaciclib) as having significant antitumor effects. Their combination was found to have the highest cytotoxicity with minimal effects on non-malignant cells, confirming stronger tumor inhibition than sorafenib (Lim JJ. et al., 2022).

The potential of HCC-PDOs in studying liver regeneration was recently reported, using PDOs generated from poorly differentiated HCC specimens injected into the right superior lobe of immunodeficient mice. The findings indicated an enhanced regenerative potential compared to animals that were not subjected to resection, thereby providing a model with greater physiological relevance than traditional models (Haak et al., 2024).

Clinical applicability for personalized therapy using HCC-PDOs is an emerging goal. For example, in the case of a 74-year-old patient with a rare neuroendocrine-differentiated HCC, PDOs were established post-surgery to guide treatment. Despite initial drug screenings, the patient’s condition deteriorated rapidly (Meier et al., 2022). Conversely, another case showed successful application of PDOs for pharmacological screening in a 55-year-old patient, leading to a significant reduction in tumor markers and size, ultimately facilitating surgical resection (He et al., 2024a).

Murine HCC organoids have also been established, particularly in transgenic mice with specific gene deletions in hepatic progenitor cells, leading to the development of aggressive HCC tumors with high metastatic potential (Zhang et al., 2023; Li et al., 2018).

Collectively, these developments underscore the importance of HCC-PDOs in precision medicine and the need for further studies to validate their clinical relevance.

### Cholangiocarcinoma patient-derived organoids (CCA-PDOs): advances in disease modeling and treatment

Research on CCA-PDOs is expanding due to their potential in disease modeling, drug testing, and personalized medicine. Given the complex nature of CCA and the lack of effective treatments, PDOs provide valuable insights. Significant studies have established protocols for generating CCA-PDOs from bile duct tissues, successfully reproducing the tumor’s histological and genetic features (Saito et al., 2019; Maier et al., 2021).

Recent analyses of PDOs identified two major iCCA subtypes—small-duct and large-duct—with distinct genetic and histological characteristics. Integrative genomic profiling revealed differences in key signaling pathways (KRAS, TGFβ, and ERBB2) enriched in large-duct tumors, underscoring the potential of organoids for personalized therapeutic strategies (Lee et al., 2023).

A case report demonstrated the utility of PDOs in guiding conversion therapy for a 59-year-old woman with advanced pCCA. After initial therapies failed, PDOs were created from a biopsy to assess drug sensitivity. Results indicated responsiveness to Gemcitabine and Cisplatin, leading to an adjusted treatment regimen that resulted in significant tumor shrinkage, making surgical resection possible. Following surgery, the patient remained disease-free at the 12-month follow-up, highlighting the effectiveness of PDOs in personalized treatment planning (He et al., 2024b).

Innovative technologies are enhancing drug screening in CCA organoids. Kinome profiling across different organoid models revealed distinct kinase activity patterns that correlated with tumor responses to specific inhibitors, suggesting a promising approach to personalized treatment strategies targeting pathways like EGFR, PDGFRβ, and MAPK (Lieshout et al., 2022).

Label-free brightfield microscopy, in conjunction with an organoid-specific image analysis pipeline, demonstrated the selective growth inhibition of iCCA-PDOs by Sorafenib, particularly in tumor cells, and identified potential applications for low-dose Sorafenib in patients with KRAS mutations (Koch et al., 2022).

Another study developed a protocol for inducing branching morphogenesis in cholangiocyte and cholangiocarcinoma organoids, providing a model for studying biliary function and pathology (Ober et al., 2023).

### Co-culture models of patient-derived organoids in liver cancer research

Despite significant advances in liver PDOs, challenges remain in replicating the complex interactions between tumors and their stroma and accurately reflecting intratumor heterogeneity.

A recent study examined the role of CAFs in HCC tumor initiation. Mice treated with diethylnitrosamine (DEN) had LGR5+ knock-in cells to model HCC. Co-culturing organoids with primary CAFs enhanced the proliferation of LGR5+ PDOs and increased tumor growth and metastasis *in vivo* (Zhang et al., 2023).

Another study developed a co-culture model of human HCC spheroids or PDX-derived organoids and endothelial cells in macroporous hydrogels. Direct co-cultures showed increased angiogenesis-related proteins and induced an inflammatory phenotype, suggesting a pro-angiogenic environment in HCC (Lim JTC. et al., 2022).

Zhou et al. (2022) established a co-culture system integrating CCA-PDOs with immune cells to evaluate immune-mediated cytotoxicity. The experiments demonstrated that T cells were the primary mediators of organoid cytotoxicity, producing effector cytokines like interferon-gamma (IFN-γ) and tumor necrosis factor-alpha (TNF-α) upon interaction with organoids. Their findings revealed patient-specific cytotoxic effects, emphasizing the importance of soluble factors in immune responses (Zhou et al., 2022).

To study tumor-stroma interactions and chemotherapy resistance, a co-culture system was created using eCCA organoids and tumor-associated macrophages (TAMs). The findings indicated that eCCA organoids co-cultured with TAMs were more resistant to chemotherapy agents, underscoring TAMs’ role in supporting tumor growth and drug resistance. This model could serve as a robust platform for personalized drug testing and understanding TAMs’ contributions to treatment mechanisms (Guo et al., 2024).

Future developments may incorporate additional tumor microenvironment (TME) cells and optimized culture conditions to enhance preclinical models.

### Organoid-on-a-chip technology in liver cancer research

The organoid-on-a-chip technology offers a promising approach to studying liver tumors and advancing drug development. This innovative technology integrates organoids with microfluidic devices, creating an environment that closely mimics *in vivo* conditions. Microfluidic devices facilitate precise control of factors like nutrient gradients, oxygen levels, and shear stress, simulating the dynamic environment of human tissues, thus offering a more physiologically relevant system than static (both 2D and 3D) cultures (Telles-Silva et al., 2022).

Recent developments have led to the creation of a microfluidic platform featuring hepatic spheroids and organoids, designed to sustain liver-specific functions through efficient nutrient and oxygen exchange. This vascular-like network enables continuous flow, closely simulating liver blood vessels. Cultured within this system, hepatic spheroids and organoids demonstrated sustained viability, preserved morphology, and liver-specific protein expression, highlighting a stable microenvironment. The organoids exhibited active liver enzymes, including critical CYP450 isoforms for drug metabolism. The platform successfully mirrored *in vivo* toxicity profiles in response to hepatotoxic drugs like acetaminophen, indicating its potential for accurate preclinical testing (Bonanini et al., 2022).

Zou et al. (2023) introduced a micro-engineered organoid-on-a-chip platform for predicting immunotherapy responses in HCC patients. This model integrates MSCs with HCC organoids to replicate key aspects of TME. Co-culturing PDOs with MSCs significantly enhanced organoid growth and the expression of tumor markers like alpha-fetoprotein and Ki67. The study assessed the platform’s utility in predicting immunotherapy responses by treating the organoid model with anti-PD-L1 antibody. The results revealed varying responses to immunotherapy, reflecting the heterogeneity observed in clinical settings. MSCs influence the immune microenvironment, promoting macrophage differentiation toward an M2 phenotype while enhancing immune cell recruitment and exhibiting immune suppression through cytokine secretion. These findings suggest that MSCs in the TME play a significant role in mediating resistance to immunotherapy, potentially explaining the variable patient responses (Zou et al., 2023).

## Discussion

**FIGURE 1:**
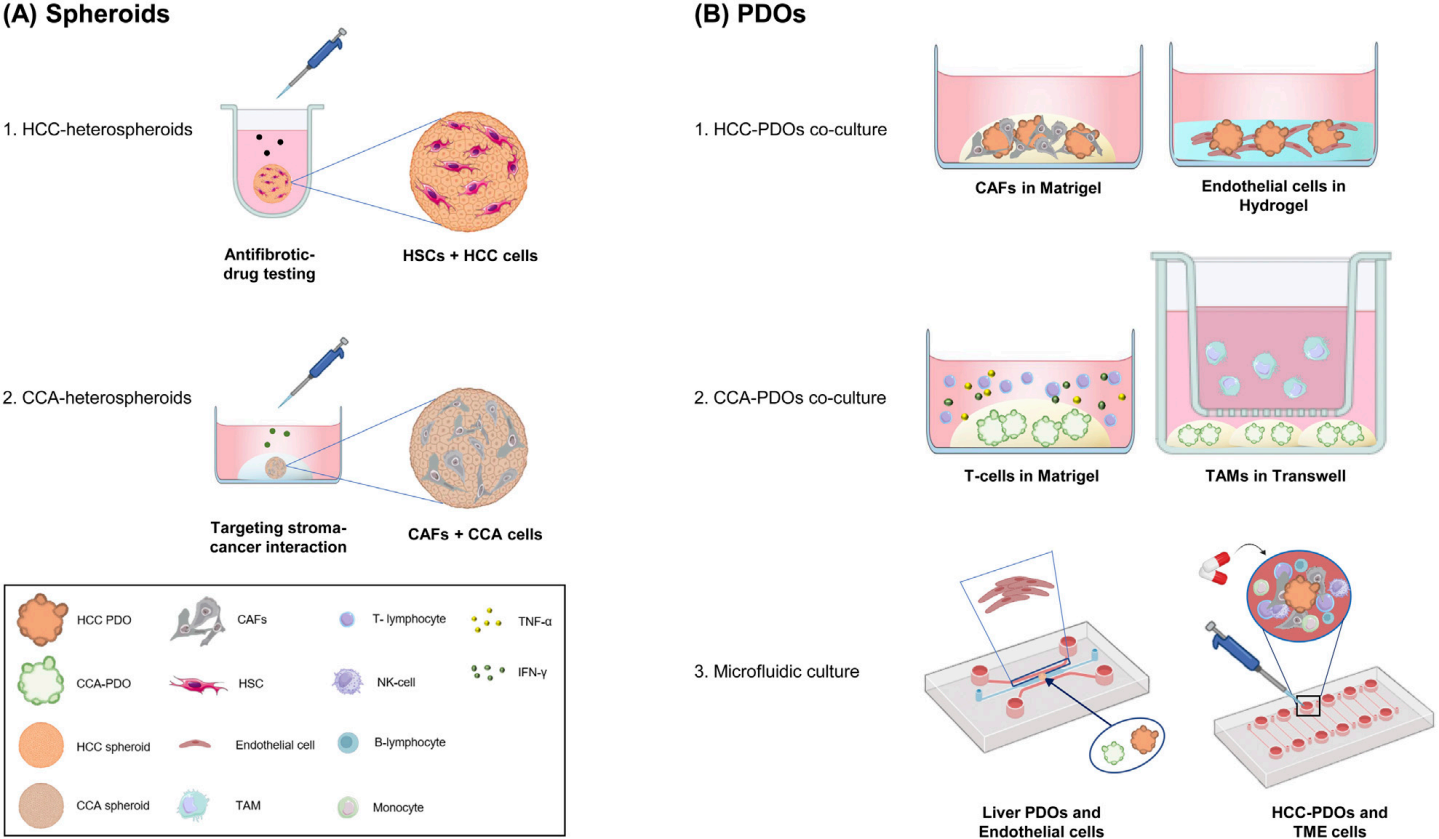
Advances in Spheroids and Organoids for Liver Cancer Research. A schematic overview of advances in the applications of spheroids and PDOs in liver cancer research. **(A)** Heterospheroids combining mesenchymal cells and cancer cells to better mimic the tumor microenvironment. The 3D cultures are used to investigate tumor-stroma interactions and test novel treatments. **(B)** PDOs: Co-cultures and microfluidic devices with PDOs and stroma components. 1) HCC-PDOs with CAFs or endothelial cells mimic the interaction between cancer cells and the TME. 2) CCA-PDOs with T-cells enable the study of tumor-immune cell interactions and checkpoint inhibitor responses; CCA-PDOs with TAMs highlighting the role of macrophages in tumor growth. 3) Organoids-on-Chip integrate organoids in a dynamic microfluidic culture, that incorporates endothelial cells and PBMCs. These systems mimic vascularization and immune surveillance, which are crucial for proving therapy efficacy. Cancer-Associated Fibroblasts (CAFs); cholangiocarcinoma (CCA); Hepatic Stellate Cells (HSCs); Hepatocellular Carcinoma (HCC); Patient-Derived Organoids (PDOs); Interferon-Gamma (IFN-γ); peripheral blood mononuclear cells (PBMCs), Tumor Necrosis Factor-alpha (TNF-α), Tumor-Associated Macrophages (TAMs); Tumor Microenvironment (TME). Figures were created with BioRender.com (free version), Microsoft Paint 3D and with Bioicons.com.

Applying spheroids and organoids in studying HCC and CCA represents a significant advancement in cancer research. These 3D models provide a more accurate representation of tumor architecture, cellular interactions, and heterogeneity than traditional 2D cell cultures. They are essential for investigating cancer biology, drug response, and resistance mechanisms. Figure 1 provides a schematic representation of the design of some recent models, along with their corresponding applications.

Despite these advancements, challenges persist, including variability in organoid cultures, the necessity for enhanced standardization, and difficulties in fully recapitulating the tumor microenvironment. Nevertheless, ongoing refinements of these models are expected to improve their clinical relevance, facilitating drug development and enhancing our understanding of cancer progression.

The development and use of spheroids and organoids in research represent a shift in biomedical sciences, especially when compared to traditional animal models. These advanced 3D cell culture systems offer significant advantages in terms of biological relevance by providing human-relevant alternatives to animal testing. They align closely with the principles of the 3Rs—Replacing, Reducing, and Refining—by substituting animal models with human-derived systems, diminishing reliance on animal studies, and refining experimental methodologies (Tosca et al., 2023). This approach allows for high-resolution insights into cellular dynamics and molecular mechanisms without the invasive techniques necessary in animal research.

These models present a promising avenue for personalizing cancer treatment, reducing reliance on animal models, and improving predictions of human-specific drug toxicity and efficacy, thus progressing liver cancer research and therapeutic innovation.

### General conclusion

Research on both spheroids and organoids has revolutionized the field of liver cancer, offering *in vitro* models that faithfully replicate the characteristics of original tumors. These models serve as powerful tools for identifying therapeutic targets, biomarkers, and effective treatments, marking a significant advance toward the realization of personalized medicine.

## Abbreviations

CCA, Cholangiocarcinoma; HCC, hepatocellular carcinoma; MSCs, Mesenchymal stromal cells; PDOs, patient-derived organoids; CAFs, cancer-associated fibroblasts; PDX, patient-derived xenograft; ICIs, immune checkpoint inhibitors; PBMCs, peripheral blood mononuclear cells; NKCEs, NK cell engagers; GPC3, glypican-3; HSCs, hepatic stellate cells; TAMs, tumor-associated macrophages; TME, tumor microenvironment.
